# A longitudinal daily diary study of stress, self-esteem fluctuation, and mood variability in adolescents

**DOI:** 10.1038/s41598-026-64789-x

**Published:** 2026-08-05

**Authors:** Christoph Borzikowsky, Maren Prignitz, Stella Guldner, Herta Flor, Frauke Nees, IMAC-Mind Consortium, Rainer Thomasius, Tobias Banaschewski, Herta Flor, Johannes Kornhuber, Michael Klein, Olaf Reis, Tanja Legenbauer, Antonia Zapf, Nicolas Arnaud, Austermann Maria, Christiane Baldus, Anne Daubmann, Léa Josette Laurenz, Sabrina Kunze, Sophie Luise Schiller, Michael Supplieth, Frauke Nees, Karl Gottfried, Stella Guldner, Sabina Millenet, Maren Prignitz, Bernd Lenz, Peter Fasching, Matthias Beckmann, Hartmut Heinrich, Verena Nadine Buchholz, Anna Eichler, Lothar Häberle, Christiane Mühle, Patrick Stelzl, Bernhard Volz, Katharina Ise, Diana Moesgen, Lucie Waedel, Martin Holtmann, Regina Herdering, Carina Maria Huhn, Laura Mokros, CoviDrug Consortium, Frauke Nees, Rabia Zohair, Karina Janson, Tobias Banaschewski, Nathalie Holz, Stella Guldner, Herta Flor, Maren Prignitz, Olaf Reis, Elisa Wandinger, Antonia Fröhlich, Emanuel Schwarz, Ersoy Kocak

**Affiliations:** 1 Institute of Medical Psychology and Medical Sociology, University Medical Center Schleswig-Holstein, Kiel University, Preußerstraße 1-9, 24105 Kiel, Schleswig-Holstein Germany; 2 Department Neuropsychology and Psychological Resilience Research, Research Group Learning and Brain Plasticity in Mental Disorders, Central Institute of Mental Health, Medical Faculty Mannheim, Heidelberg University, Mannheim, Germany; 3 Institute of Child and Adolescent Psychiatry and Psychotherapy, Central Institute of Mental Health, Medical Faculty Mannheim, Heidelberg University, Mannheim, Germany; 4 Institute of Medical Psychology, Faculty of Medicine, Ludwig Maximilians University of Munich, Munich, Germany; 5 University Medical Center Hamburg–Eppendorf, Hamburg, Germany; 6 Friedrich-Alexander-Universität Erlangen, Nürnberg, Germany; 7 Catholic University of Applied Sciences, Cologne, Germany; 8 Department of Psychiatry, Neurology & Psychosomatics in Childhood and Adolescence, University Medicine Rostock, Rostock, Germany; 9 Ruhr-University Bochum, Bochum, Germany; 10 Department of Psychiatry and Psychotherapy, Central Institute of Mental Health, Medical Faculty Mannheim, University of Heidelberg, Mannheim, Germany; 11 Hector Institute for Artificial Intelligence in Psychiatry, Central Institute of Mental Health, Medical Faculty Mannheim, Heidelberg University, Mannheim, Germany

**Keywords:** Adolescents, Emotional stability, Stress experience, Self-esteem, Mediation analyses, Health care, Psychology, Psychology

## Abstract

Adolescence represents a developmental phase marked by profound and rapidly shifting emotional states. While some adolescents exhibit pronounced, day-to-day mood variability, others remain comparatively stable. Although emotional fluctuation has long been considered a core feature with implications for health and well-being, the determinants underlying these substantial individual differences remain insufficiently understood. To address this, we conducted a longitudinal study using daily app-based assessments, aiming to explore whether perceived stress and self-esteem dynamics help explain why some adolescents show greater mood variability than others. We asked N = 70 adolescents to rate their self-esteem, positive, and negative mood once per day for 15 days. For data analyses, we used path analytic mediation modeling. Fluctuation of self-esteem (FSE) significantly predicted both fluctuation of positive mood (FPM; *b* = 0.282, 95% CI [0.101, 0.464], *p* = .0.002) and fluctuation of negative mood (FNM; *b* = 0.395, 95% CI [0.147, 0.644], *p* = 0.002) indicating that greater variability in self-esteem is associated with greater mood fluctuation. Moreover, perceived stress (PS) significantly predicted FSE (*b* = 0.057, 95% CI [0.012, 0.102], *p* = 0.013), suggesting that higher perceived stress was associated with greater self-esteem fluctuation. Significant indirect effect emerged for PS on FPM (*b* = 0.016, 95% CI [0.003, 0.030], *p* = 0.019) and on FNM (*b* = 0.023, 95% CI [0.002, 0.043], *p* = 0 .028) via FSE, confirming a mediating role of self-esteem fluctuations. This study showed that perceived stress was associated with fluctuations in self-esteem, which were related to mood variability among adolescents. These findings suggest that stress reduction could be explored as a potential avenue for future interventions.

## Introduction

Adolescence is widely recognized as a critical developmental transition into adulthood^1^. This period involves navigating new responsibilities, building independence, and forming long-term habits and identity. Adolescents face multitude of challenges, including physical growth, cognitive development, and adjustments within the family and social environment^2^. One of the most affected domains during this period is the emotional sphere. It is well known that adolescents experience strong and rapid daily mood changes^3^, often ranging from extreme elation to profound sadness. However, the degree of these emotional fluctuations varies widely. While some adolescents show intense variability, others remain more emotionally stable and withdrawn^4^.

Since the COVID-19 pandemic, the urgency to better understand emotional stability in adolescents has become more and more important. Several studies have documented increased emotional problems among adolescents during lockdowns, driven by isolation, disrupted routines, and a lack of peer contact^5–7^. These developments highlight the need to investigate the underlying mechanisms and processes of emotional (in)stability, in order to develop effective strategies and interventions for supporting adolescents in times of crisis and beyond.

### Emotional fluctuations and its consequences

While increased emotional sensitivity is a normative characteristic of adolescent development, enduring or pronounced emotional fluctuations (i.e., a high amplitude of emotions) may constitute a risk factor for psychological maladjustment. Past research found that untreated emotional instability in adolescents increases the risk for a range of long-term mental health issues, including depressions^8,9^, anxiety disorders^10,11^, psychosomatic complaints^9^, social difficulties^12^, and a lower quality of life^2^. Thus, there is an urgent need to detect and treat emotional fluctuations in young people as early as possible. However, this requires a thorough identification and understanding of the underlying factors.

### Risk factors for emotional fluctuations

Up to now, past research has identified several risk factors for excessively high emotional instability in adolescence, including genetic predispositions, socioeconomic status, personality traits, parental stress, and most importantly, perceived personal stress, and self-esteem^13,14^. In the present study, we focus on perceived stress and self-esteem as potential factors influencing adolescents’ mood fluctuations, which are discussed in more detail in the following chapters.

#### The role of perceived stress

Perceived stress is defined as the amount of stress a person feels in response to life’s demands, rather than the actual number or intensity of stressors^15^. Adolescents often face major transitions (e.g., academic pressure, social relationships, family conflict, and identity exploration) that can elevate perceived stress. High perceived stress during adolescence plays a significant role in both the continuity and the co-occurrence of externalizing and internalizing problems^16,17^. It is linked to emotional disturbances, including irritability, mood swings, anxiety, and depression^18^. Based on these studies, it seemed that high levels of perceived stress could overwhelm adolescents’ coping resources, leading to increased vulnerability to emotional problems. For this reason, we focus on this factor in the present study.

#### The role of self-esteem

Self-esteem is defined as a person’s overall evaluation of their worth^19^. In adolescence—a period marked by identity formation, social comparison, and heightened sensitivity to peer evaluation—self-esteem becomes especially crucial. High self-esteem is a protective factor that helps adolescents cope with challenges, failures, and social pressures^20,21^. It promotes resilience and adaptive coping strategies^22^. At the same time, low-self-esteem is consistently associated with poor emotion regulation, increased anxiety, depression, loneliness, social withdrawal, and even aggressive or risky behavior^23,24^. Low self-esteem leads to negative evaluations of daily life events that are interpreted as reflections of personal failure and to engage in negative self-talk, both of which heighten emotional distress. Accordingly, this study places additional emphasis on this factor.

#### The dynamic interplay between perceived stress and self-esteem

Some findings also point to reciprocal effects between perceived stress and self-esteem^25^. That is, both factors are negatively correlated and are associated with emotional well-being^26^. On the one hand, adolescents with low self-esteem tend to perceive stressful situations as more threatening and feel less capable of handling them^27^. Thus, low self-esteem has been linked to higher levels of perceived stress, and high perceived stress has been associated with emotional disturbances what can lead to or worsen mental health problems. In the same direction points a study by Byrne et al.^28^ who reported that perceived stress mediates the relationship between self-esteem and emotional distress.

On the other hand, a more recent study suggests that chronically perceived stress can undermine self-esteem^29^. This study investigated the psychobiological mechanism underlying the indirect association between emotional intelligence and self-esteem through perceived stress in military personnel. Key findings revealed that perceived stress links the association between emotional intelligence and self-esteem, demonstrating that adaptive emotional regulation reduces subjective stress perception, which in turn protects self-worth. As it seemed, intense or prolonged stress or anxiety can permanently impair self-esteem so that both directions are possible: High perceived stress can undermine self-esteem, and low self-esteem can amplify the perception of stress^16^.

### The present study

While emotional instability is a typical entity of adolescence, its severity and impact vary, and in some cases may lead to long-term mental health challenges if left unaddressed. As it seemed, perceived stress and self-esteem are central for understanding emotional disturbances in adolescents because they influence how young people interpret, experience, and respond to life’s challenges. Low self-esteem heightens vulnerability, high perceived stress amplifies emotional strain, and their interaction can trigger or intensify depression, anxiety, and other emotional difficulties.

However, the exact underlying mechanism linking perceived stress, self-esteem, and mood fluctuations remain underexplored—especially in real-life, longitudinal settings. While there is large agreement on the importance of perceived stress and self-esteem for adolescents’ mood fluctuations, there is still disagreement about their exact roles. Past research identified perceived stress as the mediator variable that is influenced by self-esteem and, in turn, influences emotional and social outcomes^30^. However, it is questionable whether the relationships between perceived stress and self-esteem might not be reversed. Therefore, identifying underlying mechanisms and factors associated with these individual differences in emotional fluctuation and their interplay is critical, but still underexplored.

To address this question and to put new empirical evidence into this discussion, we used data of a longitudinal study, aiming to explore whether perceived stress and self-esteem dynamics help explain why some adolescents show greater mood variability than others. In particular, we tested self-esteem (rather than perceived stress, as investigated in previous studies) as the mediator variable of interest.

**Fig. 1:**
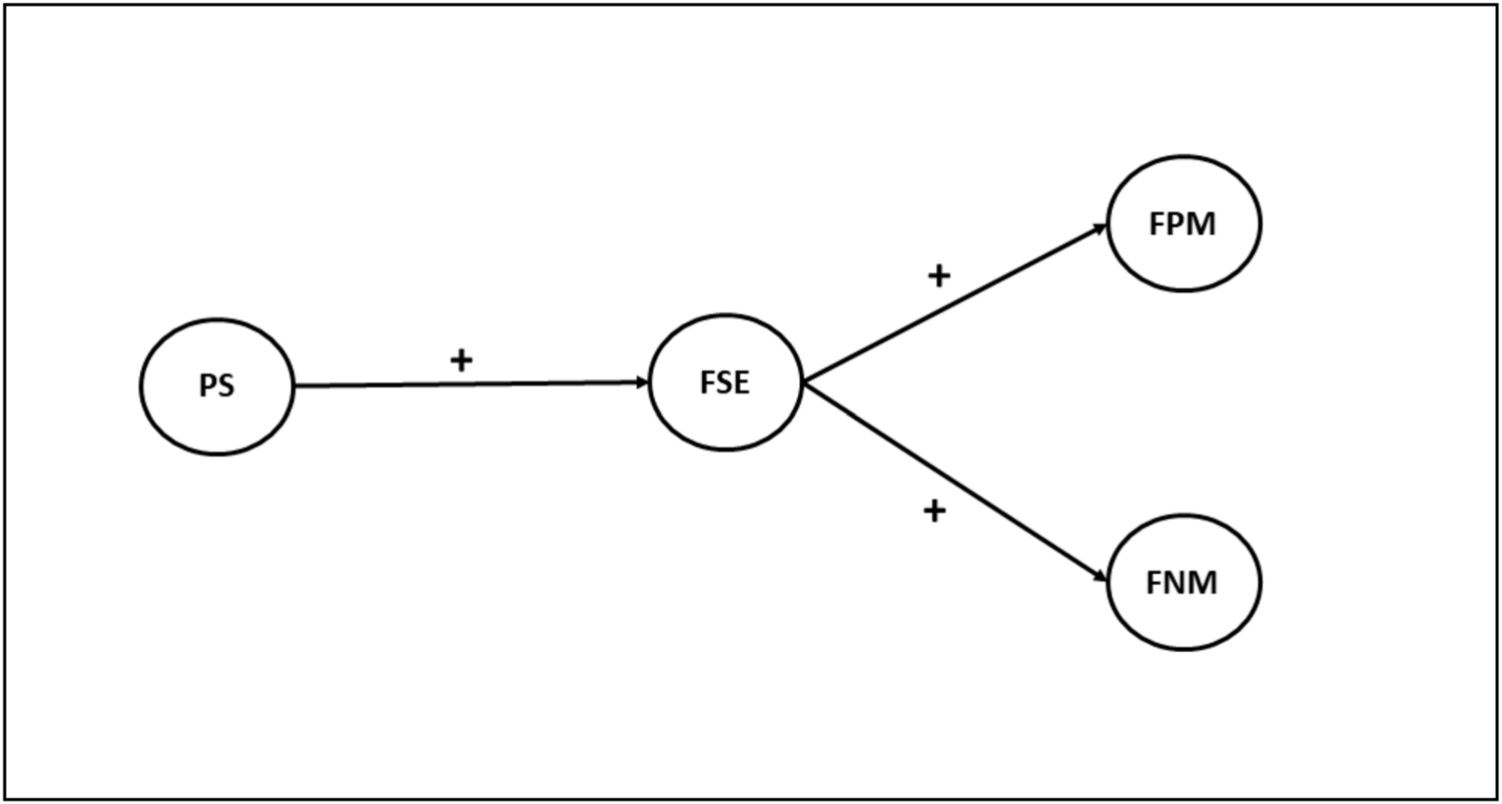
Hypothesized model with assumed associations between the variables perceived stress (PS), fluctuation of self-esteem (FSE), fluctuation of positive mood (FPM), and fluctuation of negative mood (FNM). Assumed positive associations are marked with “+”.

Therefore, in our *Fluctuation-Hypothesis*, we assumed that high self-esteem fluctuation should predict high mood fluctuation (i.e., positive (H_1a_) and negative (H_1b_) mood). Second, we expected in our *Stress-Hypothesis* that high perceived stress should predict high fluctuation in self-esteem (H_2_). Third, the expected effect of perceived stress on mood fluctuation (i.e., positive (H_3a_) and negative (H_3b_) mood) should be mediated by fluctuation in self-esteem (*Mediation-Hypothesis*, see Fig. 1).

## Methods

### Study background

We analyzed data from the cohort of work package two (WP 2) of the IMAC-Mind research project (Improving Mental Health and Reducing Addiction in Childhood and Adolescence through Mindfulness: Mechanisms, Prevention and Treatment, BMBF 01GL1745B). The aim of this project was to improve the prevention and treatment of children and adolescents with substance use disorders and associated mental disorders.

Participants were drawn from two school grade levels, which typically correspond to ages 14 and 16 years at the time of assessment. The selection of participants aged 14 and 16 years was guided by the aim to capture two distinct stages of mid-to-late adolescence, which are characterized by important developmental transitions. These age groups were chosen a priori to allow for comparisons across adjacent adolescent cohorts within the longitudinal design.

The data analyzed in the present study were collected within the framework of this longitudinal intervention study. However, the present analyses are based exclusively on baseline and pre-intervention measurements obtained prior to the onset of any intervention components for increasing mindfulness. The analysis and design of the current study were not pre-registered.

### Procedure

Data collection took place between August 2018 and October 2020. At the beginning, participants visited our lab in Mannheim, Germany, and were asked to underwent a comprehensive test battery of self-report questionnaires, neurobehavioral tasks and structured interviews. At this face-to-face meeting, participants were also introduced to an Ecological Momentary Assessment (EMA)^31^ which is a smartphone app-based tool. The advantage of EMA was to capture participants’ experiences and feelings in context as daily snapshots and, thus, prevented biased post-hoc memories. Participants were asked to answer the questions presented in the EMA-app every day on their smartphones over a time period of 15 days. For this reason, participants carried a study phone (Nokia 5 with Android version 7.1.1 Nougat) during the data collection period (for a detailed description of the study procedure see^32^.) Participants were instructed to not skip questions and push-messages were sent out every day as a gentle reminder for participation. Participants’ EMA-data was transmitted instantly to our secure local servers and saved according to German data security protection (DSGVO). To guarantee complete anonymity, participants’ questionnaires were coded with id-numbers. This procedure was in accordance with the German law of data protection and ethical standards.

### Instruments

#### Daily positive and negative mood

Emotional instability in the present study was operationalized as aggregate within-person variability across the 15-day period, quantified using the absolute sum of pairwise day-to-day differences in affect ratings (positive and negative mood). This approach captured the extent of moment-to-moment variability rather than sustained deviations from baseline.

For capturing adolescents’ daily mood within the EMA-app, we used the *Positive and Negative Affect Schedule* (PANAS)^33^. Over a time period of 15 days, we measured participants’ positive mood with 10 items (e.g., “Right now I feel happy.”). The same number of items was used to measure participants’ negative mood (e.g., “Right now I feel bad.”). For each item, participants were asked to indicate their current feelings on a 7-point Likert scale from “1 = not at all” to “7 = very” every day. All items were positively formulated (i.e., no reversed coding). Because we were interested in daily mood fluctuations, mean values were calculated across the 10 items for positive (negative) mood for each day so that higher values correspond with higher positive (negative) mood.

#### Daily self-esteem

Within the EMA-app, participants’ daily self-esteem was assessed with the *Rosenberg Self-Esteem Scale* (RSES)^19^. For the 15-days-time period, participants answered every day four items on a 7-point Likert scale from “1 = I completely agree” to “7 = strongly disagree” (e.g., “Currently I am satisfied with myself”). Two items were positively and two items negatively formulated. After reversed scoring of the two negatively formulated items, mean values for each day were calculated across the four items so that higher values correspond with higher self-esteem.

#### Perceived stress

During the institute assessment, participants rated their perceived stress experienced in the last year with the *Adolescent Stress Questionnaire* (ASQ)^28^. The ASQ was introduced with the following statement: “Below you will find some statements about things and situations that you might find stressful. Please indicate how much each item has stressed you during the past year”. After that, participants answered 30 items with a 5-point Likert-scale from “1 = not stressful at all” to “5 = very stressful” (e.g., “Struggles between you and your parents”). All items were positively formulated (i.e., no reversed coding). A mean value was calculated across the 30 items so that a higher value corresponds with a higher level of perceived stress. Since we were interested in long-term perceived stress, this questionnaire was only used once at the beginning of the study and not every day.

In the present study, we followed and tested the idea that perceived stress is the primary predictor and not a mediator. The reason for that is grounded in the longitudinal study design: We measured perceived stress (via ASQ) during the institute assessment at the beginning of the study (baseline). Besides sociodemographic covariates, all other variables were later measured in the EMA app. This longitudinal sequence of measurements supports the interpretation of perceived stress as a predictor rather than a mediator.

#### Sociodemographic variables

The paper-and-pencil questionnaire at baseline contained several questions about participants’ sociodemographic background as well. For our later analyses, we used the variables gender (0 = male, 1 = female), age (0 = 14 years old, 1 = 16 years old), and intelligence (intelligence quotient, IQ, measured with the CFT 20-R)^34^ as covariates in our models. Gender was included as a covariate due to well-documented sex differences in emotional stability (neuroticism), with women typically scoring lower than men^35^. Age was included as a covariate because emotional stability has been shown to vary systematically across the lifespan, with emotional stability generally increasing and neuroticism decreasing across adulthood^36^. Intelligence (IQ) was included as a covariate because cognitive ability has been shown to relate to individual differences in personality and emotional functioning^37^. We used these three variables as our background model and controlled for their effects on parameter estimation.

### Data preparation

Before analyzing empirical data, we did plausibility checks for all variables and created three new variables that were relevant for later hypothesis testing. For positive mood, negative mood, and self-esteem, we calculated the absolute sum of pairwise differences over the 15-day period, resulting in three fluctuation scores: fluctuation of self-esteem (FSE), fluctuation of positive mood (FPM), and fluctuation of negative mood (FNM). Consequently, values equal or close to zero represent stability of mood and self-esteem over the 15-days-period, respectively. In contrast, values much higher than zero represent fluctuation across time.

We used pairwise differences between adjacent days rather than mean values and standard deviations over the 15-day period because aggregate measures may be disproportionately affected by extreme observations (i.e., very high or very low values) and can obscure short-term day-to-day variability. Pairwise differences provide a more direct and robust characterization of temporal changes between consecutive assessments.

### Statistical methods

For calculating descriptive statistics, we used *IBM SPSS Statistics* for Windows (Version 29.0.2.0)^38^. For categorical variables, we calculated absolute (*n*) and relative frequencies as percentages (*%*). For metric variables, we calculated mean values (*M*) and standard deviations (*SD*). Path analyses and mediation analyses were performed in the software *Mplus 7* for Windows^39^.

For testing the *Fluctuation-Hypothesis* (H_1_), we calculated a linear regression model with FPM as the outcome variable and FSE as the main predictor variable (H_1a_). In this model, the variables age, gender, and intelligence served as additional covariates. We also included the variable PS as covariate into this model to control for its effect. The second part of the *Fluctuation-Hypothesis* (H_1b_) was tested in the same way but this time FNM served as the outcome variable. For testing the *Stress-Hypothesis* (H_2_), we calculated a linear regression model with FSE as the outcome variable and PS as the main predictor variable. Age, gender, and intelligence served as additional covariates. For testing the first part of our *Mediation-Hypothesis* (H_3a_), we used PS as the main predictor variable, FPM as the outcome variable, and FSE as the mediator variable. The variables age, gender, and intelligence served as additional covariates. We repeated the same procedure for testing the second part of our *Mediation-Hypothesis* (H_3b_) and used this time FNM as the outcome variable.

For all predictor variables, we reported the unstandardized regression weight (*b*), the corresponding standard error (*SE*), the 95% confidence interval (*CI*), the standardized regression weight (*β*), and the p-value (*p*). For all analyses, we used the Full Information Maximum Likelihood (FIML) method for handling missing values. P-values smaller than 0.05 were regarded as statistically significant.

## Results

### Descriptive statistics

The analyzed sample consisted of *N* = 70 German adolescents who were either 14 years (*n* = 26, 37.1%) or 16 years (*n* = 38, 54.3%) old (*n* = 6, 8.6% missing values). In this sample, *n* = 35 (50.0%) people were female, *n* = 29 (41.4%) people were male, and *n* = 6 (8.6%) people didn’t indicate their gender. The IQ in the sample was *M* = 105.35 (*SD* = 12.78, *n* = 62; Min = 86.00, Max = 138.00). Missing values for age and gender (n = 6) resulted from incomplete administrative or questionnaire records at baseline. Missing IQ data (n = 8) were due to participants being absent during baseline testing.

Descriptive mean values were *M* = 67.65 for PS (*SD* = 19.66, *n* = 60; Min = 34.00, Max = 108.00), *M* = 4.42 for FSE (*SD* = 3.16, *n* = 69; Min = 0.00, Max = 16.44), *M* = 4.88 for FPM (*SD* = 2.03, *n* = 69; Min = 0.55, Max = 10.67), and *M* = 4.70 for FNM (*SD* = 2.55, *n* = 69; Min = 0.61, Max = 13.60).

### Path analysis

#### Fluctuation-hypothesis

As shown in the upper part of Table 1, empirical data supported the first part of the *Fluctuation-Hypothesis* (H_1a_): FSE predicted FPM positively (*b* = 0.282, 95% CI [0.101, 0.464], *p* = 0.002) meaning that greater self-esteem fluctuation was associated with greater positive mood fluctuation (R^2^ = 0.271). The same was true for age (*b* = 1.081, 95% CI [0.215, 1.948], *p* = 0.014) what reflected the emotional up and downs with advancing age. Furthermore, we found that FSE predicted FNM positively (*b* = 0.395, 95% CI [0.147, 0.644], *p* = 0.002; see lower part of Table 1). Thus, greater self-esteem fluctuation was associated with greater negative mood fluctuation as well (R^2^ = 0.345).

**Table 1:** Results for FPM and FNM (Fluctuation-Hypothesis).

Model 1	Outcome FPM	*b*	*SE*	95% CI	*β*	*p*
No.	Predictor variable					
0	Intercept	2.171	2.147	[− 2.038; 6.380]	–	0.312
1	FSE	0.282	0.093	[0.101; 0.464]	0.440	0.002**
2	PS	− 0.007	0.015	[− 0.036; 0.022]	− 0.069	0.630
3	Age	1.081	0.442	[0.215; 1.948]	0.263	0.014*
4	Gender	0.305	0.487	[− 0.650; 1.260]	0.075	0.532
5	IQ	0.011	0.019	[− 0.028; 0.049]	0.066	0.589

Model 2	Outcome FPM	*b*	*SE*	95% CI	*β*	*p*
No.	Predictor variable					
0	Intercept	2.807	3.164	[− 3.395; 9.009]	–	0.375
1	FSE	0.395	0.127	[0.147; 0.644]	0.490	0.002**
2	PS	0.015	0.018	[− 0.020; 0.051]	0.118	0.400
3	Age	− 0.295	0.496	[− 1.267; 0.676]	− 0.057	0.551
4	Gender	0.702	0.507	[− 0.291; 1.696]	0.138	0.166
5	IQ	− 0.010	0.027	[− 0.063; 0.043]	− 0.051	0.706

*N* = 70; Model 1: *R*^2^ = 0.271; Model 2: *R*^2^ = 0.345.

*b* unstandardized regression weight, *SE* standard error, *95% CI [b]* 95% confidence interval for unstandardized regression weight, *β* standardized regression weight, *p* p-value, *FPM* fluctuation of positive mood, *FNM* fluctuation of negative mood, *FSE* fluctuation of self-esteem, *PS* perceived stress, *Age* age of adolescents (0 = 14 years old, 1 = 16 years old), *Gender* gender of adolescents (0 = male, 1 = female), *IQ* intelligence of adolescents.

*p < 0.05; **p < 0.01.

As a sensitivity analysis, we repeated the analyses without including any covariates (i.e., without gender, age, and IQ). We found the same results (FSE predicted FPM positively (*b* = 0.299, 95% CI [0.099, 0.499], *p* = 0.003; R^2^ = 0.201); FSE predicted FNM positively (*b* = 0.414, 95% CI [0.161, 0.666], *p* = 0.001; R^2^ = 0.317). In summary, the key results stayed the same.

#### Stress-hypothesis

As presented in Table 2, we found that PS positively predicted FSE (*b* = 0.057, 95% CI [0.012, 0.102], *p* = 0.013). Thus, empirical evidence supported our assumption that higher perceived stress was associated with greater self-esteem fluctuation (R^2^ = 0.159).

**Table 2:** Results for the FSE (Stress-Hypothesis).

Model 3	Outcome FSE	*b*	*SE*	95% CI	*β*	*p*
No.	Predictor variable					
0	Intercept	3.968	3.423	[− 2.742; 10.677]	–	0.246
1	PS	0.057	0.023	[0.012; 0.102]	0.358	0.013*
2	Age	0.581	0.698	[− 0.787; 1.948]	0.091	0.405
3	Gender	0.522	0.828	[− 1.101; 2.145]	0.083	0.528
4	IQ	− 0.038	0.029	[− 0.095; 0.019]	− 0.153	0.195

*N* = 70; Model 3: *R*^2^ = 0.159.

*b* unstandardized regression weight, *SE* standard error, *95% CI [b]* 95% confidence interval for unstandardized regression weight, *β* standardized regression weight, *p* p-value, *FSE* Fluctuation of Self-Esteem, *PS* Perceived Stress, *Age* Age of Adolescents (0 = 14 years old, 1 = 16 years old), *Gender* Gender of Adolescents (0 = male, 1 = female), *IQ* Intelligence of Adolescents.

*p < 0.05.

As a sensitivity analysis, we repeated the analyses without including any covariates (i.e., without gender, age, and IQ). We found the same results (*b* = 0.054, 95% CI [0.013, 0.096], *p* = 0.010; R^2^ = 0.115). In summary, the key results stayed the same.

#### Mediation-hypothesis

In line with our assumptions, the direct effect (*b* = -0.007, *p* = 0.630) from PS to FPM was not significant (see upper part of Table 3). However, and against our expectations, the total effect was not significant as well (*b* = 0.009, *p* = 0.516). However, decomposing the total effect revealed the indirect effect (path a × path b) as a significant pathway (*b* = 0.016, 95% CI [0.003, 0.030], *p* = 0.019), with both path a (i.e., from PS to FSE: *b* = 0.057, 95% CI [0.012, 0.102], *p* = 0.013) as well as path b (i.e., from FSE to FPM: *b* = 0.282, 95% CI [0.101, 0.464], *p* = 0.002) being significant (R^2^ = 0.271). Observing a not significant total effect in combination with a significant indirect effect can be interpretated in terms of a partial mediation. Thus, presented empirical evidence could partially support the first part of our *Mediation-Hypothesis* (H_3a_).

**Table 3:** Results for FPM and FNM mediation analyses (Mediation-Hypothesis).

Mediation 1	Outcome FPM	*b*	*SE*	95% CI	*β*	*p*
No.	Mediation pathways					
1	Direct effect (PS → FPM)	− 0.007	0.015	[− 0.036; 0.022]	− 0.069	0.630
2	Path a (PS → FSE)	0.057	0.023	[0.012; 0.102]	0.358	0.013*
3	Path b (FSE → FPM)	0.282	0.093	[0.101; 0.464]	0.440	0.002**
4	Indirect effect (path a × path b)	0.016	0.007	[0.003; 0.030]	0.157	0.019*
5	Total effect (direct + indirect)	0.009	0.014	[− 0.018; 0.036]	0.088	0.516

Mediation 2	Outcome FPM	*b*	*SE*	95% CI	*β*	*p*
No.	Mediation pathways					
1	Direct effect (PS → FNM)	0.015	0.018	[− 0.020; 0.051]	0.118	0.400
2	Path a (PS → FSE)	0.057	0.023	[0.012; 0.102]	0.358	0.013*
3	Path b (FSE → FNM)	0.395	0.127	[0.147; 0.644]	0.490	0.002**
4	Indirect effect (path a × path b)	0.023	0.010	[0.002; 0.043]	0.175	0.028*
5	Total effect (direct + indirect)	0.038	0.018	[0.002; 0.073]	0.294	0.037*

*N* = 70; Mediation Model 1: *R*^2^ = 0.271; Mediation Model 2: *R*^2^ = 0.345.

*b* unstandardized regression weight, *SE* standard error, 95% CI [*b*] 95% confidence interval for unstandardized regression weight, *β* standardized regression weight, *p* p-value, *FPM* fluctuation of positive mood, *FNM* fluctuation of negative mood, *FSE* fluctuation of self-esteem, *PS* perceived stress, *Age* age of adolescents (0 = 14 years old, 1 = 16 years old), *Gender* gender of adolescents (0 = male, 1 = female), *IQ* Intelligence of Adolescents.

*p < 0.05; **p < 0.01.

As a sensitivity analysis, we repeated the analyses without including any covariates (i.e., without gender, age, and IQ). As before, the direct effect from PS to FPM was not significant (*b* = -0.007, *p* = 0.644) as well as the total effect (*b* = 0.010, *p* = 0.446). However, the indirect effect (path a × path b) revealed again as a significant pathway (*b* = 0.016, 95% CI [0.002, 0.030], *p* = 0.024), with both path a (i.e., from PS to FSE: *b* = 0.054, 95% CI [0.013, 0.096], *p* = 0.010) and path b (i.e., from FSE to FPM: *b* = 0.299, 95% CI [0.099, 0.499], *p* = 0.003) being significant (R^2^ = 0.201). In summary, the key results stayed the same.

As expected, the direct effect from PS to FNM was not significant (*b* = 0.015, *p* = 0.400, see lower part of Table 3). However, in line with our expectations, the total effect (direct + indirect effect) was significant (*b* = 0.038, 95% CI [0.002, 0.073], *p* = 0.037). Focusing on the significant indirect effect (*b* = 0.023, 95% CI [0.002, 0.043], *p* = 0.028), showed a significant path a (i.e., from PS to FSE: *b* = 0.057, 95% CI [0.012, 0.102], *p* = 0.013) as well as a significant path b (i.e., from FSE to FNM: *b* = 0.395, 95% CI [0.147, 0.644], *p* = 0.002; R^2^ = 0.345). In summary, the significant total effect with a non-significant direct effect and a significant indirect effect can be interpretated as a full mediation. Thus, the presented results could confirm the second part of our *Mediation-Hypothesis* (H_3b_).

As a sensitivity analysis, we repeated the analyses without including any covariates (i.e., without gender, age, and IQ). As before, the direct effect from PS to FNM was not significant (*b* = 0.015, *p* = 0.395). However, the total effect (direct + indirect effect) revealed as significant again (*b* = 0.038, 95% CI [0.004, 0.072], *p* = 0.030). Focusing on the significant indirect effect (*b* = 0.023, 95% CI [0.001, 0.044], *p* = 0.039), showed a significant path a (i.e., from PS to FSE: *b* = 0.054, 95% CI [0.013, 0.096], *p* = 0.010) and a significant path b (i.e., from FSE to FNM: *b* = 0.414, 95% CI [0.161, 0.666], *p* = 0.001; R^2^ = 0.317). In summary, the key results stayed the same.

## Discussion

### Determinants of emotional stability in adolescents

The present study aimed to investigate why some adolescents experience stronger emotional fluctuations in their daily life than others. Based upon previous research, we focused on perceived stress and self-esteem as two psychological factors that may be associated to variability in adolescents’ emotional states. To address the underexplored links and mechanisms between these two risk factors, we used data of a longitudinal study, aiming to investigate in a statistical mediation model possible associations between perceived stress, self-esteem dynamics, and adolescents’ mood variability. In particular and in line with recent studies in the military context (see^29^), we tested in this study self-esteem as the mediator variable of interest. We used daily app-based assessments to collect ecologically valid data in in real-life, longitudinal settings and explored the relationships between adolescents’ general stress perceived in the last year, their daily self-esteem, and their daily positive and negative mood. For this reason, we formulated and evaluated three hypotheses using path-analytic mediation modeling.

First, we hypothesized and confirmed that fluctuations in self-esteem are positively associated with fluctuations in both positive and negative mood. This finding supports our *Fluctuation-Hypothesis* and aligns with existing literature that self-esteem plays a central role for emotional and behavioral problems in adolescents^23,24^. Notably, the effect was somewhat stronger for negative mood than for positive mood, which may reflect the asymmetric impact of low self-worth on emotional well-being.

Second, we found evidence for our *Stress-Hypothesis*: Higher levels of perceived stress were associated with greater variability in daily self-esteem. This supports the notion that stress can undermine self-perception during adolescence, as also reported in prior studies^16^. Therefore, we recommend to parents, teachers, and other practioners to decrease adolescents’ perceived stress in order to evolve stable and non-volatile self-perceptions for their children.

Third, we tested our *Mediation Hypothesis* which combined the two preceding models. Results showed that fluctuating self-esteem significantly mediated the relationship between perceived stress and fluctuations in both positive and negative mood. The findings suggest that self-esteem might partially account for the association between stress and emotional fluctuations in adolescents.

Taken together, the empirical findings of the present study underscore the importance of self-esteem fluctuation in the association between perceived stress and mood variability during adolescence. Enhancing adolescents’ self-esteem may therefore represent a critical target for interventions aimed at reducing emotional fluctuation. Future studies should investigate the causal pathways between stress, self-esteem, and emotional fluctuations and their interplays.

### Implications

The empirical findings presented in this paper offer fruitful and valuable implications for both research and applied psychology. In particular, the results of this longitudinal study highlight the importance of stress reduction interventions as a means of stabilizing self-esteem and, indirectly, promoting emotional stability among adolescents. Because perceived stress is modifiable (e.g., via mindfulness or cognitive-behavioral coping strategies, stress management techniques^40^, and self‐esteem can be improved (e.g., via positive self-concept interventions)^41^, these findings may have implications for intervention development for reducing emotional disturbances in adolescents as they target both external appraisals (stress perception) and internal resources (self-esteem).

Given that untreated emotional instability in adolescents can be associated to a range of negative outcomes in adulthood—including depression, anxiety, and reduced quality of life^42,43^—early intervention is critical. Our results provide preliminary evidence that strategies aimed at reducing adolescents’ perceived stress may contribute to improvements in self-perception and emotional resilience.

From a clinical perspective, stabilizing adolescents’ self-esteem (e.g., through social skills training, positive self-reflection exercises, or strengths-based counseling)^44,45^ may represent a potential target for interventions aimed at reducing emotional dysregulation. These findings provide preliminary support for exploring interventions focused on emotion regulation, stress management, and self-concept strengthening as possible means of promoting adolescent mental health.

### Research limitations

Despite the strengths of this study, several limitations should be acknowledged: First, the analyzed study sample consisted of N = 70 adolescents, which is relatively small and did not allow for more advanced statistical analyses such as structural equation modeling (SEM). While path analysis provides valuable insights into directional relationships among observed variables, SEM would allow the inclusion of *latent* constructs which improve the model’s robustness. Thus, future research should aim to replicate our findings using a larger sample and SEM-based frameworks.

Second, the 15-days observation period may not have been sufficient to capture the full scope of emotional fluctuations, especially for adolescents whose mood variability unfolds more gradually. Longer study durations could yield more nuanced insights into the temporal dynamics of stress, self-esteem, and emotional regulation. Moreover, future studies should explore the directionality of effects between stress and self-esteem in greater detail, ideally using cross-lagged panel designs.

Third, perceived stress was only measured once at the beginning of this study what does not reflect daily perceived stress, but rather a more general perception of stress. Maybe daily perceived stress could have had a more direct, immediate, and stronger impact on adolescents’ mood fluctuations, but this cannot be determined using the ASQ.

## Conclusion

This study aimed to investigate why some adolescents experience stronger emotional fluctuations in their daily life than others. Based upon previous research, we focused on perceived stress and self-esteem as two psychological factors that may be associated to variability in adolescents’ emotional states. To address the underexplored links and mechanisms between these two risk factors, we conducted a longitudinal study, aiming to explore in a path-analytic mediation model whether perceived stress and self-esteem dynamics could predict adolescents’ mood variability. In a sample of N = 70 adolescents we found that higher perceived stress was associated with greater self-esteem fluctuation (i.e., a more volatile self-perception), which in turn was associated with mood variability among adolescents (i.e., increased amplitude of emotions).

These findings highlight the possible role of perceived stress in influencing self-esteem, which could be associated with subsequent mood variability in adolescents. Interventions targeting perceived stress could be associated with improvements in self-esteem, emotional stability, and psychological well-being during adolescence. Identifying this mechanism in the presented mediation model, could probably help to prevent adolescents from the far-reaching negative consequences of undiscovered emotional problems.

## Data Availability

This data has not been made publicly available. Anonymized study data and statistical codes to analyses may be made available on request to the corresponding author.
